# Prospective evaluation of complications associated with orthosis and prosthesis use in canine patients

**DOI:** 10.3389/fvets.2022.892662

**Published:** 2022-07-29

**Authors:** Sydney Rosen, Felix Michael Duerr, Lindsay Hochman Elam

**Affiliations:** Department of Clinical Sciences, Colorado State University College of Veterinary Medicine and Biomedical Sciences, Fort Collins, CO, United States

**Keywords:** orthotic, prosthetic, rehabilitation, orthopedics, dog, cranial cruciate ligament disease

## Abstract

**Introduction:**

The use of orthoses and prostheses is expanding in veterinary medicine. However, research evaluating the efficacy and complications of these devices in veterinary patients is limited. The primary objective of this study was to prospectively determine the complications and outcomes associated with custom orthosis and prosthesis use in the canine patient.

**Materials and Methods:**

This was a prospective, clinical trial that followed patients for 12 months following device fitting. Owner-perceived complications, clinical metrology instruments, and objective gait analysis were used as outcome measures at various time points. The patients were grouped into the following four major categories: Patients with a carpal orthosis, patients with a stifle orthosis, patients with a tarsal orthosis, and patients with a prosthetic device.

**Results:**

Forty-three patients were included in the study. Thirty-nine out of 43 patients (91%) experienced at least one complication, with 7/7 (100%) prosthesis patients experiencing at least one complication. At least one skin complication was reported for the following patient groups during the first 3 months of use: 8/14 (58%) stifle orthoses, 9/10 (90%) carpal orthoses, 6/10 (60%) tarsal orthoses, and 4/7 (58%) prostheses. Patient non-acceptance of the device was identified in 2/15 (14%) stifle orthoses, 1/10 (10%) tarsal orthoses, and 4/7 (55%) prostheses. One out of 15 (7%) stifle orthoses, 4/10 (40%) carpal orthoses, 4/10 (40%) tarsal orthoses, and 1/7 (15%) prostheses experienced mechanical device problems necessitating repair. The majority of patients with carpal and stifle orthoses showed improvement on objective gait analysis in percent body weight distribution of the affected limb between baseline and the most recent follow-up without the device donned: 83% (*n* = 6) of patients with carpal orthoses, 100% (*n* = 11) of patients with stifle orthoses. None of the patients with tarsal orthoses showed a similar improvement (0%; *n* = 4).

**Discussion and conclusion:**

Three major complications associated with canine orthosis and prosthesis use were identified in this study as follows: Skin complications (abrasions, loss of hair, and sores), mechanical device problems, and patient non-acceptance of the device. Owners should be notified of these potential complications prior to pursuing orthoses or prostheses as a potential treatment option. Although clinical improvement was noted in the majority of patients with stifle and carpal pathology, given the lack of a control group, it is unknown how much of this improvement can be attributed to the orthoses.

## Introduction

The use of orthoses and prostheses in companion animals has become increasingly popular in veterinary medicine (1). Orthoses have a variety of orthopedic applications and can serve to restrict, control or assist with motion, and/or function as a protective device (1). Bertocci et al. found that ~51% of owners were interested in non-surgical intervention for the treatment of cranial cruciate ligament disease (CCLD) due to misgivings about surgical intervention, while 29% sought an orthosis due to the cost associated with surgical intervention (2). Prostheses enable use of an incomplete limb resulting from either amputation or a congenital defect (1, 3).

Despite this emerging popularity and variety of applications, research evaluating the efficacy and complications of orthoses and prostheses in veterinary patients is limited. There has been multitudinous research in human medicine on the topic, but the significant differences in the anatomy and gait of veterinary patients warrants research specific to companion animals.

The available veterinary research suggests that orthoses may play a role in decreasing lameness and pain associated with several conditions in companion animals (4–8). Tomlinson et al. retrospectively reviewed canine patients with carpal ligament instability and found return to normal function for 79% of patients with significantly improved lameness scores in patients with carpal ligament instability that was refractory to cage rest. Hart et al. found that 88% of dogs wearing stifle orthoses for CCLD had mild to no lameness at the conclusion of the study based on owner assessment (5). However, the previously mentioned studies relied on subjective outcome measures of lameness exclusively, utilizing visual lameness scoring and client surveys to determine degree of lameness and overall outcome. Bertocci et al. showed improved joint stifle mechanics associated with application of an orthosis for CCLD compared to a cranial cruciate ligament deficient stifle in a computer model (6). Case et al. demonstrated improvement in a dog with Type 2c common calcanean tendinopathy treated with both an orthosis and mesenchymal stem cell transplantation (7). This case report did utilize force plate gait analysis as an objective outcome measure. A retrospective study by Carr et al. utilized a pressure sensitive walkway to evaluate a carefully selected group of 10 dogs fitted with a stifle orthosis for CCLD. This study showed an improvement of total pressure index of 5.1% in the affected limb after 90 or more days when compared to baseline (8). Given the concurrent use of other treatments and lack of appropriate control groups, determination of the effectiveness of the orthotic alone is unclear.

In addition to the lack of clear knowledge of the benefits of these devices, one of the major complications associated with the application of veterinary orthotics or prosthetics (VOP) is skin sores. Mechanical forces applied to the skin by an orthosis may result in loss of integrity to the skin (9). At the time of writing, no study has prospectively evaluated complications associated with the application of canine orthoses.

The available socket prosthesis research suggests that owner satisfaction and quality of life with these devices is high, despite considerable complication rates (3, 10, 11). In a study by Wendland et al., 96% of surveyed owners indicated that they would elect to utilize a prothesis as a treatment option again and 89% of patients were shown to have acceptable to full function based on author-defined clinical outcome scoring criteria (3). In a study by Phillips et al., 8/12 dogs fitted with a socket prosthesis had a good outcome overall and quality of life remained good or excellent in 10/12 dogs (10). In a study by Carr et al., 50% of surveyed owners reported that the patient's mobility had improved with the application of the prosthesis and 37.5% of surveyed owners reported no change in the patient's mobility (11). A retrospective case series on intraosseous transcutaneous amputation prostheses (ITAP) for limb-sparing in malignant neoplasia reported that all dogs had pain-free limb function following application (12).

Several studies have reported on complications associated with socket prosthesis use, including development of sores, prosthesis failure (device breaking), and poor patient compliance in using the prosthesis (3, 10, 11). The primary reported complication with ITAP in canine patient is endoprosthesis fracture which was managed with replacement of the ITAP. There were no reported skin complications reported with ITAP in canine patients (12). However, all these studies were retrospective in nature and no prospective studies regarding use of socket or intraosseous prostheses in canine patients have been published to date. Other concerns with ITAP include the higher cost, need for specialized equipment/implants, possibility of complications with the internal fixation, and the implant-skin interface.

While orthoses have been suggested to provide a valuable, less invasive alternative for certain musculoskeletal conditions, and prostheses have been suggested to be a viable replacement for incomplete limbs, more objective data is needed to aid veterinarians and owners in the decision process when considering these novel treatment options. The primary goal of this study was to prospectively determine the type and incidence of complications associated with application of orthoses and prostheses in canine patients.

## Materials and Methods

Participation in the study was offered to all canine patients that presented to the Colorado State University Veterinary Teaching Hospital (CSU-VTH) over a 2-year period (2018–2020) for lameness or mobility concerns related to musculoskeletal pathology that was deemed to benefit from a custom VOP. There were no other specific inclusion criteria established, such as requirements regarding patient age or size. Dogs who were diagnosed with concomitant neurologic conditions that affected their gait or dogs that were non-compliant/aggressive (unlikely to tolerate device application or gait analysis) were deemed ineligible for the study. Study visits were planned at device fitting (baseline), 3, 6, and 12 months after fitting. Veterinary examinations were performed at these visits to subjectively assess patient progress, check for development of comorbidities, and determine if adjustments to the devices were warranted. Incentives for participation included waived examination and some diagnostic fees at the study visits, a 50% discount for one device, and an additional $600 reimbursement (approximately the second half of the cost of the device) for the completion of all study visits and surveys to encourage a continued participation.

The study protocol was approved by the CSU-VTH Clinical Review Board (VCS #2018-171). Patient care, including pain management and physical therapy, was dictated by the residents and faculty members of the Orthopedic Medicine and Mobility service at the James L. Voss Veterinary Teaching Hospital and decisions related to their care were made independent of the study.

### Surveys

#### Online survey

An online survey (Table 1) was developed to collect information regarding device complications, owner-reported outcomes and satisfaction, device use, concurrent therapies, and changes to the patient's daily life. Surveys were sent by email monthly for 12 months, with the first survey sent 1 month after orthosis or prosthesis fitting via the Survey Monkey online platform (www.surveymonkey.com). If a patient was fitted for bilateral devices simultaneously, the owner received one survey for both devices. If a patient was fitted for bilateral devices at different time points, the owner received a separate survey for each device.

**Table 1:** Online survey questions and answer options.

Q1- During the last month, have there been any changes in your dog's health, environment or medication regime?	•Yes (please specify the changes) •No, everything is unchanged
Q2- On average, during the last month, how often did your dog wear the brace on a daily basis?	•Not at all •A few minutes per day •A few hours per day •Almost all day (but not at night) •Almost all day and night
Q3- During the last month, did you adhere to the brace wearing schedule suggested by your veterinarian?	•Yes •No, my dog wore the device more often than recommended •No, my dog wore the device less often than recommended •My veterinarian did not suggest a wearing schedule •Other (please specify)
Q4- Has your dog received any form of rehabilitation (physical therapy) during the last month?	•Yes, our dog had at least one session with a rehabilitation (physical therapy) specialist and we have performed rehabilitation at home •Yes, our dog had at least one session with a rehabilitation (physical therapy) specialist •Yes, we have performed rehabilitation (physical therapy) ourselves at home •No •Other (please specify)
Q5- Overall, during the last month, how much do you think your dog benefited from the brace?	*(Please use the slider below to select how beneficial the brace was for your dog)*
Q6- Overall, during the last month, how active was your dog?	*(Please use the slider below to select your dog's activity level)*
Q7- Overall, during the last month, how happy was your dog?	*(Please use the slider below to select your dog's happiness level)*
Q8- Overall, during the last month, how satisfied are you with the brace as a treatment for your dog's disease?	*(Please use the slider below to select your satisfaction level with the brace)*
Q9- During the last month, have there been any complications (other than skin sores) associated with the brace?	•Yes (please specify the complication/s) •No, everything is fine
Q10- During the last month, did your dog develop any skin sores, skin irritation, or other wounds from wearing the brace?	•Yes •No
Q11- Please describe the skin sore, irritation, or wound.	*Text box*
Q12- Did you have your dog assessed by a veterinarian for the skin sore, irritation, or wound?	•Yes •No •I have not yet, but I plan to •Other (please specify)

*All questions were required to be answered by the owners apart from questions 11 and 12, which were only asked of the owner if they reported a skin complication in question 10. Questions 5–8 were answered with a slider bar with available options between 0 and 100*.

Only patients with complete surveys from at least the first 3 months were included in data analysis. The survey responses were evaluated by one author (SR) for consistency and accuracy. Whether a patient experienced a complication (for example, a skin complication) was determined based on whether a free-response description of that complication was provided.

Skin complication severity was categorized based on the description provided by the owners. Minor skin complications were defined as owner-described loss of hair, irritation, or small sores. Major skin complications were defined as owner-described bleeding, large sores, or signs of infection. An owner description that was vague or unclear was labeled “unknown.” Two of the authors (SR and FD) reviewed the descriptions and agreed on categories for the descriptions provided. Other device complications were noted if they were mentioned in any of the free-response sections of the survey.

#### Client specific outcome measures

Owners completed the activity component of the CSOM questionnaire using methodologies previously published (13). Owners were asked to pick up to five time and place specific problematic activities and grade them on a scale of 1–5 (1 = no problem, 2 = a little problematic, 3 = quite problematic, 4 = severely problematic, and 5 = impossible). The questionnaire was collected at baseline, 3, 6, and 12 months after fitting and completed in dependent fashion, with the grades established at the previous timepoints available to the owner for comparison. The average of these scores was taken to determine a combined score at each timepoint, with a decrease in score indicating improvement. The owners did not complete the behavior component of the CSOM questionnaire.

### Objective gait analysis

The objective gait analysis using a pressure sensitive walkway (Tekscan HRV Walkway 6 VersaTek system, Tekscan Inc., South Boston, MA) analysis system was collected at baseline, 3 months after fitting, 6 months after fitting, and 12 months after fitting. The patients were weighed at each visit prior to collection of OGA data, both without their device and with their device so accurate weights could be used for gait data with and without the device. The patients were evaluated at a walk, pace, or trot, based on patient self-selected gaits at baseline and recommended restrictions for each patient based on diagnosed pathology. For example, all patients with common calcaneal tendinopathy were evaluated at a walk. The OGA data was collected using a previously described protocol (14, 15). Three valid trials were obtained in both directions (six total trials) without lateralization of the head, stepping off the pressure sensitive walkway, or pulling on the leash. A trial was also deemed invalid if the patient would not utilize the affected limb (was non-weight bearing), as kinematic variables of the non-weight bearing limb would not be able to be assessed (15). If the patient was only compliant walking in one direction at baseline, the trials were only obtained walking in that direction at all follow-up visits. The trials were only considered valid if the velocity of the dog was within 0.3 m/s of the previous trials. The video recorded during the gait analysis data collection was reviewed to ensure that the program had appropriately labeled each foot placement.

Percent body weight distribution (%BW) of the limb fitted with the VOP was calculated by dividing the peak vertical force of the limb fitted with the VOP by the peak vertical force of all four limbs throughout the gait cycle and multiplying that number by 100 (16). The %BW was then averaged from the six valid trials. If six valid trials were unable to be obtained due to patient compliance, the average of the valid trials was calculated. If the patient had bilateral devices, %BW was recorded for both affected limbs and included in statistical analysis, regardless of when the devices were fitted. Improvement of %BW was defined as any increase in %BW on the limb fitted with the VOP.

For orthoses patients, OGA was collected both with and without the device donned. OGA trials without the device donned were only collected in patients with orthopedic injuries that would not be harmed by the patient ambulating without the device.

### Statistical analysis

For statistical analysis, CSOM and OGA data from the baseline visit and most recent follow-up visit available for each patient was utilized. If the patient did not have a baseline CSOM or any follow-up CSOM questionnaires, they were excluded from CSOM data analysis. Only patients with both baseline and follow-up data for OGA (with and/or without the device donned) were included in %BW data analysis.

If an owner reported, or the examining veterinarian identified, the development of a comorbidity unrelated to device use and this comorbidity was determined to likely affect the patient clinically, the data for the affected variables were not included in data analysis. If the comorbidity was temporary, the data was only omitted from analysis for the times during which the comorbidity was present.

The available online survey data was evaluated for instances of complications throughout the 12 months and types of complications were recorded. The skin complications were analyzed in two groups, those occurring within the first 3 months of the study and those occurring between months 4 and 12.

For statistical analysis of complications, the sore severity category “unknown” was included in the minor category. Analysis was performed based on individual patients as opposed to each instance of skin complication. If a patient experienced at least one major skin complication, they were analyzed within the major skin complication group, otherwise they were analyzed within the minor skin complication group.

Following completion of data collection, patients were divided into four major device groups for statistical analysis. These included patients with a carpal orthosis (CO), patients with a stifle orthosis (SO), patients with a tarsal orthosis (TO), and patients with a prosthetic device (PD). The data from patients who did not fall within one of these four device groups were not included in the statistical analysis.

Fisher's Exact test was utilized to determine associations between categorical variables, including percent of owners discontinuing use of device with device group, patient non-acceptance with device group, patient non-acceptance with discontinuation of the device, mechanical device problems with device group, skin complications in the first 3 months with device group, skin complications between 4 and 12 months with device group, sore severity (for both first 3 months and between months 4 and 12 months) with device group, and whether a veterinary evaluation occurred with sore severity (for both first 3 months and between 4 and 12 months). Fisher's Exact test was utilized due to small counts in some categories. Spearman correlation was used, due to non-normally distributed data, to determine the association between the number of skin complications and the number of veterinary evaluations reported by the owners in the survey specifically for these skin complications. The Kruskal–Wallis test was used to determine if there was a difference between device categories in terms of number of mechanical device failures. A one-way ANOVA F-test was used for comparison of differences in %BW and CSOM scores between device categories. When the F-test revealed a *p* < 0.05, Tukey adjusted pairwise comparisons were performed.

## Results

Sixty-one patients were enrolled in the study and 43 patients with at least the first 3 months of complete online surveys were included for analysis of all available data (Table 2). Fourteen patients were fitted with stifle orthoses, with one patient fitted for two stifle orthoses ~6 months apart, resulting in 15 SO analyzed. The remaining categories included 10 CO, 10 TO, and 7 PD. One patient was fitted with a forelimb device for partial brachial plexus avulsion and did not fall into the four major device categories. All SO were prescribed for treatment of cranial cruciate ligament disease (CCLD). Five out of 10 (50%) CO were prescribed for treatment of carpal hyperextension. The other CO were prescribed for various conditions including carpal instability, carpometacarpal instability, deep digital flexor myotendinopathy, antebrachiocarpal luxation, and digital hyperextension. Six out of 10 (60%) TO were prescribed for treatment of common calcanean tendinopathy. The other TO were prescribed for various conditions including tarsal instability, medial collateral ligament instability, postoperative support for superficial digital flexor tendon repair with deep digital flexor tendon imbrication, and postoperative talar fracture fixation support. Two out of 7 (29%) PD were prescribed following amputation for neoplasia (osteosarcoma and soft tissue sarcoma), 2 out of 7 (29%) were prescribed for congenital defects or following amputation secondary to congenital defects, out of 7 (29%) were prescribed for patients following traumatic amputation or amputation secondary to trauma, and 1 out of 7 (15%) was prescribed for a patient with unknown cause of partially missing limb.

**Table 2:** Patient signalment, diagnosis, and device group.

**No**	**Breed**	**Age (years)**	**Sex**	**Diagnosis**	**Device group**
1	Australian Shepherd	6	MC	Brachial plexus avulsion	Full forelimb orthotic device
2	Australian Cattle Dog	1	FS	Antebrachiocarpal luxation	CO
3	Border Collie	9	MC	Deep digital flexor myotendinopathy	CO
4	Australian Shepherd	6	MI	Carpal instability	CO
5	Anatolian Shepherd	9	MC	Digital hyperextension	CO
6	Mixed Breed	6	FS	Lateral carpometacarpal instability	CO
7	Labrador Retriever	11	MI	Carpal hyperextension	CO
8	Mixed Breed	7	FS	Carpal hyperextension	CO
9	Mixed Breed	7	MC	Carpal hyperextension	CO
10	Border Collie	10	MC	Carpal hyperextension	CO
11	German Shepherd	12	FI	Carpal hyperextension	CO
12	Standard Poodle	8	MC	Cranial Cruciate Ligament Disease	SO
13	Standard Poodle	8	MC	Cranial Cruciate Ligament Disease	SO
14	Staffordshire Terrier	9	FS	Cranial Cruciate Ligament Disease	SO
15	Great Pyrenees	5	MC	Cranial Cruciate Ligament Disease	SO
16	Mixed Breed	9	FS	Cranial Cruciate Ligament Disease	SO
17	Mixed Breed	5	MC	Cranial Cruciate Ligament Disease	SO
18	Golden Retriever	9	FS	Cranial Cruciate Ligament Disease	SO
19	Mixed Breed	8	MC	Cranial Cruciate Ligament Disease	SO
20	St. Bernard	6	FS	Cranial Cruciate Ligament Disease	SO
21	Staffordshire Terrier	7	FS	Cranial Cruciate Ligament Disease	SO
22	Mixed Breed	7	FS	Cranial Cruciate Ligament Disease	SO
23	Golden Retriever	13	FS	Cranial Cruciate Ligament Disease	SO
24	Catahoula Leopard Dog	12	FS	Cranial Cruciate Ligament Disease	SO
25	Labrador Retriever	12	MC	Cranial Cruciate Ligament Disease	SO
26	Mixed Breed	8	FS	Cranial Cruciate Ligament Disease	SO
27	Great Dane	9	FS	Osteosarcoma (amputation)	PD
28	Border Collie	1	MC	Congenital deformity	PD
29	Mixed Breed	1	MC	Missing distal forelimb (unknown if congenital or traumatic)	PD
30	Dachshund	2	MC	Traumatic amputation	PD
31	Labrador Retriever	7	FS	Soft Tissue Sarcoma (amputation)	PD
32	German Shorthaired Pointer	2	FS	Congenital deformity (amputation)	PD
33	Labrador Retriever	2	FS	Amputation secondary to trauma	PD
34	Labrador Retriever	8	FS	Common Calcaneal Tendinopathy	TO
35	Labrador Retriever	10	FS	Common Calcaneal Tendinopathy	TO
36	German Shorthaired Pointer	9	MC	Common Calcaneal Tendinopathy	TO
37	Labrador Retriever	7	MC	Common Calcaneal Tendinopathy*	TO
38	Labrador Retriever	11	FS	Common Calcaneal Tendinopathy	TO
39	Labrador Retriever	7	MC	Common Calcaneal Tendinopathy*	TO
40	Labrador Retriever	7	MC	Tarsal Instability	TO
41	Mixed Breed	2	MI	Postoperative Fracture Fixation	TO
42	Labrador Retriever	8	FS	Medial Collateral Ligament Instability	TO
43	German Shepherd	3	MC	Postoperative digital flexor tendon repair	TO

*FS, female spayed; FI, female intact; MC, male castrated; MI, male intact; CO, carpal orthosis; SO, stifle orthosis; PD, prosthetic device; TO, tarsal orthosis*.

*Bilateral devices fitted simultaneously.

The median number of months of completed online survey responses for all patients was 10, with owners completing online surveys between 3 and 12 months out of 12 possible months. Four patients were unable to complete the study following humane euthanasia, three of which were orthoses patients that were euthanized following the development of disease processes unrelated to the orthopedic condition that resulted in prescription of the device. One postoperative amputation prosthesis patient was euthanized following pulmonary metastasis of osteosarcoma. Two SO became clinical for CCLD in the opposite limb during the study. One SO developed suspected idiopathic vestibular disease, which improved after about 2 months per the owners. One CO developed carpal hyperextension of the opposite limb during the study.

Patients in multiple groups discontinued use of the device prior to veterinarian instruction, including 4 out of 15 (27%) SO, 1 out of 10 (10%) CO, 1 out of 10 (10%) TO, and 1 out of 7 (15%) PD (Table 3). Explanations for discontinuation of use included owner-perceived improvement, mechanical device problems, patient non-acceptance of device, transition to an alternate product, or development of a comorbidity eliminating activities requiring the device or affecting use of the device. There was no evidence of an association between device group and proportion of patients who stopped wearing the device prior to veterinary instruction (*p* = 0.76).

**Table 3:** Explanations and timeline for discontinuation of device use in seven patients prior to veterinary instruction.

**Explanation**	**Device group**	**Patient number** **(Table 1)**	**Month discontinued**
Transition to alternate product	CO	5	6
Owner perceived improvement	SO	24	1
Owner perceived improvement	TO	38	6
Mechanical device problems	SO	15	7
Patient non-acceptance	SO	20	10
Comorbidity	SO	23	7
Comorbidity	PD	27	5

Thirty-nine out of 43 (91%) patients experienced at least one complication (skin complication, mechanical problem, and/or patient non-acceptance of device), with 7/7 (100%) prosthesis patients experiencing at least one complication. At least one skin complication was reported for 8 out of 14 (58%) SO, 9 out of 10 (90%) CO, 6 of 10 (60%) TO, and 4 out of 7 (58%) PD during the first 3 months of use (Table 4). There was no evidence of an association between device group and proportion of patients with reported skin complications in the first 3 months (*p* = 0.3283). Twenty out of 41 (49%) patients experienced only minor skin complications in the first 3 months, while 7 out of 41 (17%) patients experienced at least one major skin complication (Table 5). There was no evidence of an association between severity of skin complications and device group (*p* = 0.1063). There was also no evidence of association between severity and seeking an evaluation by a veterinarian for the skin complication in the first 3 months (*p* = 0.2040). There was evidence of a moderate correlation between number of skin complications in the first 3 months and the number of evaluations by a veterinarian for the skin complication (*r* = 0.59, *p* = 0.0013).

**Table 4:** Percentage of patients experiencing at least one skin complication by device group.

**Device group**	**First 3 months**	**Months 4 and 12**
SO	58% (8/14)	62% (8/13)
CO	90% (9/10)	50% (5/10)
TO	60% (6/10)	23% (2/9)
PD	58% (4/7)	43% (3/7)
Total	66% (27/41)	46% (18/39)

**Table 5:** Skin complication severity by device group in first 3 months.

**Device group**	**Minor**	**Major**	**Total**
SO	6	2	8
CO	8	1	9
TO	2	4	6
PD	4	0	4
Total	20	7	27

At least one skin complication was reported for 8 of 13 (62%) SO, 5 of 10 (50%) CO, 2 of 9 (23%) TO, and 3 of 7 (43%) PD between months 4 and 12 after fitting (Table 4). There was no evidence of an association between device group and proportion of patients with reported skin complications in those months (*p* = 0.3607). Sixteen out of 39 (41%) of patients experienced only minor skin complications between months 4 and 12 after fitting, while 5 out of 39 (13%) patients experienced at least one major skin complication in those months. There was no evidence of an association between severity of skin complications and device group (*p* = 0.7354). There was no evidence of an association between skin complication severity and seeking an evaluation by a veterinarian for the skin complication between months 4 and 12 (*p* = 1). There was no evidence of a significant correlation between number of skin complications between months 4 and 12 and the number of evaluations by a veterinarian for the skin complication (*r* = 0.33, *p* = 0.1436).

Eleven owners reported mechanical device problems (Table 6), which included minor problems such as screws coming loose, expected wear and tear of replaceable items such as the hook and loop tape, tread, and padding, and various device components coming detached. These problems required repair by the owner, the prescribing veterinarian, or manufacturing company (depending on extent of damage) at least once, with seven owners reporting mechanical device problems between 2 and 4 times. One out of 15 (7%) SO, 4 out of 10 (40%) CO, 4 out of 10 (40%) TO, and 1 out of 7 (15%) PD experienced mechanical device problems. The full forelimb orthotic device also experienced mechanical device problems. There was no evidence of an association found between device group and mechanical device problems (*p* = 0.1110). There was also no evidence of a difference in device groups in number of mechanical problems reported (*p* = 0.1288).

**Table 6:** Mechanical problems by device group and average number of times reported (among those reporting mechanical device problems).

**Device experiencing mechanical problems**	**Average number of times reported**
Full forelimb orthotic device	4
SO	1
CO	2
TO	1.5
PD	2

Seven patients were non-accepting of their device, as indicated by owner reports of the patient chewing on the device, resistance to device application, or refusal to utilize the limb with the device donned (Table 7). Non-acceptance of the device was identified with 2 out of 15 (14%) SO, 1 out of 10 (10%) TO, and 4 out of 7 (55%) PD. Non-acceptance was not reported with CO. There was evidence of an association between device group and lack of device acceptance (*p* = 0.0179), with PD having the highest rate of non-acceptance. There was no association between patient non-acceptance and no longer using the device (*p* = 0.3178).

**Table 7:** Non-acceptance types by patient group.

**Non-acceptance type**	**Number of SO**	**Number of CO**	**Number of TO**	**Number of PD**
Chewing on device	1	0	1	0
Resistance to device application	1	0	0	2
Refusal to utilize limb	0	0	0	2

Objective gait analysis at baseline and follow-up with the device donned and doffed were performed on all patients in which it was clinically appropriate and would not exacerbate their condition gaiting sans device. With the device donned (67%), of CO showed improvement, 8 out of 8 (100%) of SO showed improvement, 10 out of 10 (100%) of TO showed improvement, and one out of 1 (100%) of PD showed improvement in %BW of the affected limb between baseline and the most recent follow-up. All other patients had a lower %BW of the affected limb with the device donned. On average, SO showed an increase in 4.0%BW ± 3.1% (*n* = 8; percent increase of 51.6% ± 77%), CO showed an increase in 2.5%BW ± 5.9% (*n* = 7; percent increase of 10.8% ± 30%), TO showed an increase in 3.2%BW ± 2.0% (*n* = 8; percent increase of 22.2% ± 17.1%), and PD showed an increase in 2.9%BW (*n* = 1; percent increase of 34.3%) of the affected limb with the device donned between baseline and the most recent follow-up in patients for which OGA data was available (Table 8). There was no evidence of a difference in device groups for magnitude of change in %BW with the device donned (*F* = 0.18, *p* = 0.9116).

**Table 8:** Change in percent body weight distribution of the affected limb between baseline and most recent follow-up with device donned.

**Device group**	**Number of patients**	**Mean change in %BW (%)**	**Standard deviation (%)**	**Minimum (%)**	**Median (%)**	**Maximum (%)**	**Median most recent follow-up visit (months)**
SO	8	4.04	3.90	0.57	1.84	11.12	7
CO	6	2.48	5.90	−5.82	3.18	10.61	6
TO	8	3.23	2.04	1.21	2.55	7.47	6
PD	1	2.87	0	2.87	2.87	2.87	7

Without the device donned, 5 out of 6 (83%) of CO showed improvement, 11 out of 11 (100%) of SO showed improvement, and 0 out of 4 (0%) of TO showed improvement in %BW of the affected limb between baseline and the most recent follow-up. All other patients had a lower %BW of the affected limb without the device donned. On average, SO showed an increase in 4.0%BW ± 2.0% (*n* = 11; percent increase of 35.1% ± 27.6%), CO showed an increase in 3.4%BW ± 2.8% (*n* = 6; percent increase of 13.9% ± 13%), and TO showed a decrease in 2.3%BW ± 2.2% (*n* = 3; percent decrease of 12.9% ± 7.5%) on the affected limb without the device donned between baseline and the most recent follow-up in patients for which OGA was available (Table 9). There was evidence of a difference in device groups for change in %BW without the device (*F* = 8.74, *p* = 0.0024). Specifically, the change in %BW of TO was different from both SO (*p* = 0.0019) and CO (*p* = 0.0085) without the device donned. Moreover, SO and CO did not show evidence of a difference without the device donned when compared to each other (*p* = 0.8621).

**Table 9:** Change in percent body weight distribution of the affected limb between baseline and most recent follow-up without device donned.

**Device group**	**Number of patients**	**Mean change in %BW (%)**	**Standard deviation (%)**	**Minimum (%)**	**Median (%)**	**Maximum (%)**	**Median most recent follow-up visit (months)**
SO	11	3.98	2.05	1.29	3.9	9.1	12
CO	6	3.37	2.84	−1.59	3.83	6.79	9
TO	3	−2.28*	2.18	−4.78	−1.28	−0.77	12

*p < 0.05 compared to other device groups.

For CSOM scoring, improvements were seen on average across device types from baseline; SO showed an average decrease in score of 1.3 ± 0.70 (*n* = 8) with a percent decrease of 45% ± 30%, CO showed a decrease of 1.0 ± 1.11 (*n* = 7) with a percent decrease of 61% ± 35%, TO showed a decrease of 0.6 ±0.96 (*n* = 8) with a percent decrease of 36% ± 42%, and PD showed a decrease of 1.7 ± 0.79 (*n* = 5) with a percent decrease of 61% ± 33%. There was no evidence of a difference in device groups for change in CSOM score (*F* = 1.75, *p* = 0.1836).

## Discussion

The primary goal of this study was to prospectively determine complications associated with orthosis and prosthesis use in canine patients. The following three major groups of complications were identified: Skin complications, mechanical device problems, and lack of device acceptance by the patient. We found that skin complications were the most common problem, with more than half of patients in all device groups experiencing at least one skin complication in the first 3 months.

The high rate of skin complications observed is consistent with the high rate observed with casting (17). However, severe skin complications made up the minority of those described in this study. This is likely due to the early detection of sores by owners since the devices are removed at least daily. However, even minor skin complications can disrupt device use while the skin heals, reducing the amount of time the patient can spend in the device. All orthosis and prosthesis patients at the CSU-VTH, including those in this study, are instructed to follow a “break-in” schedule in an attempt to reduce skin complication occurrence. This schedule involves a slow escalation in the number of hours the patient wears the device per day over the course of several weeks. This is obviously only feasible if the patient is not required to wear the device throughout the day for 24 h, e.g., for postoperative support of a tendon repair. Skin complications may occur, despite this break-in period, due to lack of owner compliance with the prescribed schedule, ineffectiveness of the prescribed schedule, the device not fitting appropriately to the limb, owners not applying the device to the limb correctly, owners not appropriately exercise-restricting patients, or simply because the skin is unable to tolerate the applied forces. Owner education may be utilized to improve owner application of the device, including providing personal instructional videos of how to apply the device and providing guide marks on the device as to appropriate tightness of device straps. This strategy was employed in the majority of the cases. Further investigation of novel materials that can reduce complications and improve fit to attenuate device-associated skin complications is warranted.

Based on the authors' clinical experience and the previously published data, skin complications (including abrasions, open sores, loss of hair, etc.) are most likely to occur during the first 2 to 3 months following device fitting (3). This informed the criteria of making completion of the first 3 months of online surveys an inclusion criterion for data analysis, as well as analysis of the sores in two groups (first 3 months and between months 4 and 12). The proportion of patients experiencing skin complications during months 4 through 12 decreased from the first 3 months in all device groups, except for SO. The cause of SO skin complications increasing after the first 3 months is unknown, but may be due to confounding variables, such as increased patient activity level.

The present study is consistent with the previous studies of VOP, as they have also described high proportions of skin complications. In a study by Hart et al., 46% of canines wearing stifle orthoses developed skin lesions (5). Wendland et al. showed a short-term prosthetic-associated complication rate of 61.7% with skin sores being the most common complication, followed by pain, swelling, and dermatitis. The long-term complication rate in that study was 19.1%, with skin sores again being the most common, followed by pain and dermatitis (3). The higher proportion of reported skin complications in the present study compared to the previous studies may be due to the prospective nature of data collection, resulting in improved accuracy of owner reporting or due to smaller sample size of this study.

The association of number of skin complications with evaluations by a veterinarian in the first 3 months may indicate to prescribing veterinarians that there will likely be additional necessary rechecks within the first 3 months of device use. The lack of association of number of skin complications with evaluations by a veterinarian between months 4 and 12 may indicate that the number of additional rechecks can decrease after the first 3 months.

The second most common complication was mechanical device issues. These instances often required repair by the veterinarians at the CSU-VTH or being sent to the manufacturer for more extensive repairs. In case of the latter, the patient was unable to utilize the device until it was shipped back. Wendland et al. found that owner satisfaction and clinical outcome scores were positively correlated with time spent in the prosthesis (3). This may indicate that mechanical device issues, in turn resulting in decreased wear by the patient, may impact both patient's clinical improvement and owner satisfaction. It is important for veterinarians to be aware of this possible complication so that owners can be prepared prior to proceeding with this treatment option. Particularly for dogs that would clinically deteriorate without their device, two devices on hand may also be recommended. This solution may be particularly relevant for patients that are expected to wear the device life-long and clinically appear to benefit from the device (e.g., prostheses).

Prosthetic device (PD) appeared to experience mechanical device issues less often than any of the orthoses. The reason for this lower rate is unknown. However, it may be possible that a lower number of articulating portions may be a contributing factor.

The third most common complication was patients not accepting the device. This was most common among PD, with patients most commonly not using the limb and walking on their three remaining legs or being resistant to device donning. Refusal to use the prosthetic limb may be related to patient acclimation to a 3-legged gait over time. However, no significant correlation has been drawn between time from limb loss to prosthesis placement and clinical outcome (3). Patient non-compliance with prostheses may also be related to the level of the defect. The level of the defect has been suggested to contribute to limitations in planes of motion and proprioceptive feedback (3). In this study, PD that did not accept their device had varying levels of defects with the most distal at the level of the mid-metatarsus. However, the small sample size of PD precludes drawing conclusions as to whether a relationship exists between level of the defect and patient non-compliance. Physical rehabilitation may be utilized to encourage use of the limb starting with habituating the patient to weight bearing and eventually improving patient proprioception and balance with the limb donned (1, 18). Studies in humans have shown improved clinical outcomes associated with rehabilitation following prothesis fitting (19). However, no positive correlation has yet been established in canine patients (3). Lack of device acceptance was not demonstrated in the canine retrospective series or in a study of four cats with ITAP (12, 20). It is possible that endoprostheses are superior to socket prosthesis in terms of device acceptance, but further research is required to adequately compare these two options.

Of the three patients wearing orthoses that were non-accepting of the device, two were reportedly chewing on their devices (one from the SO group and one from the TO group), with one resulting in mechanical device issues requiring repair. These did not appear to be solely connected to patient acclimation to the device in this study, as destruction with one patient occurred during the first month of wear, while the other occurred during the seventh month of wear. Patient destruction of the device may be addressed by only donning the device when owner supervision is available. However, this would likely decrease time spent in the device, which may result in a decreased clinical improvement and owner satisfaction.

The third patient that did not accept the device was in the SO group. This patient was resistant to device donning and refused to stand or ambulate following device donning. This behavior began during the sixth month of wearing the orthosis and continued through the twelfth month. Physical rehabilitation and positive reinforcement training may be methods of addressing this complication, but no data was collected regarding response to interventions for the patient in this study.

It is important for veterinarians to be aware of these three possible major complications, given the high incidence, in order to adequately educate clients. The potential consequences and resolutions of these complications, such as additional veterinary evaluations, device repairs, physical rehabilitation, and/or obtaining a backup device, will increase the total cost of treatment. Hart et al. showed that financial considerations were the third-most cited reason for pursuing a stifle orthosis over surgical intervention (2). Therefore, it is imperative that owners be aware of the potential financial implications of these complications prior to electing to use orthoses or prostheses as a treatment option.

Moreover, it may be indicated for veterinarians to discuss discontinuation of the device in cases where animals are not accepting of the device, especially in cases where the patient is severely resistant to device application. Interestingly, there was no association between patient non-acceptance of the device and owners discontinuing device use prior to veterinary instruction in this study. Only one owner cited patient non-acceptance of the device as the reason for entirely discontinuing use. One owner with a patient who was non-accepting of the device discontinued use but cited the development of bursitis as the reason for discontinuing use. This may indicate that owners are reluctant to discontinue treatment in cases of non-acceptance and the quality of life of the animal may need to be assessed. Additionally, patient chewing of the device may pose a safety risk if pieces of the device are ingested and cause a gastrointestinal mechanical obstruction.

As the outcome parameter for gait analysis, %BW was chosen since Kano et al. showed that %BW was most accurate of kinetic and temporospatial gait parameters within a heterogenous group of dogs when gait velocity is controlled for (16). The observed improvement in %BW with the device donned may indicate that the use of these devices provided a benefit to the patients' diseases or alternatively simply patient acclimation to the device. Conzemius et al. suggest a 5% improvement in ground reaction forces as a guideline for what can be considered clinically important in dogs with osteoarthritis (15). While the patient population studied differs from this proposed guideline, the data support a possible beneficial effect on the disease process given that the average improvement (percent increase) in %BW was >5% in all groups with the device donned. The SO and CO groups also showed a >5% improvement without the device donned in patients for which OGA data was available, which supports a beneficial effect on the disease process. In contrast, the TO group showed a negative change in %BW without the device in patients for which OGA data was available. The differences in change of %BW with the device donned and without may also be explained by alterations in ground force reaction caused by immobilization created by the device. A study by Murakami et al. showed that the level of constraint created by device affects the ground reaction force pattern (21). Additionally, Torres et al. showed that the application of a stifle orthosis affected the kinematics for all joints and planes of motion at a walk and trot (22). This may specifically explain the noteworthy discrepancy between the improvement in TO with the device donned and without, as the tarsal devices induced a variable level of constraint based on the injury. Overall, it is worthwhile noting that there is a lack of knowledge regarding interpretation of ground reaction forces in patients with VOP devices with varying underlying pathology and this data should therefore be interpreted with caution.

This study is not able to attribute improvements in %BW to the use of the orthoses, as there were no control groups associated with each device. Similar improvement may have occurred over time in many of these patients regardless of device use. A study by Wucherer et al., for example, showed that 64% of overweight CCLD patients managed with pain medication, weight loss, and physical therapy alone showed improved quality of life and lameness after 1 year (23). There have been no studies evaluating outcome of orthoses that have utilized control groups to establish significant improvement in limb function in animals fitted with devices compared to animals not fitted with devices (4, 7, 8). Thus, the attributable benefit of these devices compared to medical management without these devices is not known.

This differs from the ability to attribute improvements in %BW with prostheses, as %BW for the affected limb without the device is generally 0, other than cases where the degree limb length discrepancy is mild, and the limb contacts the ground. Thus, any improvement in %BW from baseline to follow-up would be attributable to device use by the patient. However, data for change in %BW was only obtained for one dog in the PD group with the device donned. This was primarily due to PD non-acceptance with utilizing the limb immediately following fitting resulting in a lack of baseline data. There was one recorded instance of the patient using their prosthesis for OGA at follow up visits following non-acceptance at fitting. However, there may have been instances that were missed as OGA was not attempted at follow-up visits in multiple PD where baseline data was not obtained.

In many CO and TO, it was determined to not be allowable for the patient to ambulate without the device in place, limiting both baseline and follow-up data collection without the device donned. For patients whose owners discontinued use of the device prior to veterinary instruction, follow-up OGA data was not able to be obtained with the device donned. This is useful in consideration for design of possible future VOP studies, as OGA data collection can be limited by these particulars.

On average, patients in all device groups showed improvement of CSOM score, indicating perceived clinical improvement in patient activities by the owner. It is important to note that the CSOM is not currently validated for use in canine patients with orthoses or prostheses. Additionally, the CSOM typically involves owners selecting patient behaviors to grade in addition to activities (13). Owners were not asked to grade behaviors in this study, as patients' behaviors were expected to change with the application of the device. However, the owners selected activities common in the patient's life, such as ability to climb stairs or ability to play with other dogs without lameness. Despite not being validated for this purpose, improvement in the CSOM score still indicates that, on average, patients were able to resume activities with decreased difficulty as perceived by the owner. It is also important to note that there was no control group, as such it is difficult to interpret owner perception (e.g., how much of the improvement is attributable to caregiver placebo effect).

There are several limitations associated with this study when it comes to the outcome assessment aspect of the study. Most importantly, as noted above, there were no control groups and injuries varied within all device groups, except for SO. Many patients received adjunctive medical therapies as prescribed by the veterinarians at the CSU-VTH, including physical therapy, shockwave therapy, joint injections, and others. Moreover, device use was not measured objectively and was inconsistent among the patients throughout the study. Thus, it is unknown whether the degree of clinical improvement can be attributed to the device or to the other treatments or time alone.

The residual limbs of PD were not uniform; amputations for patients were not performed using a standardized approach, patients had a variety of congenital anatomic defects, one patient had a traumatic amputation, and the history of one patient was unknown. The variability in residual limbs may have confounded patient improvement measures. Additionally, there were no inclusion criteria regarding patient breed or size. Thus, patient conformation may have introduced another confounding variable to our analysis.

The sample sizes were also small within each device group, despite 2 years of enrollment in the study. The sample sizes for OGA collection were reduced further due to censorship and the OGA clinical considerations with devices described above. The small sample sizes limited the power associated with our analysis, which reduced the probability of detecting true differences.

The online survey was developed by the authors and was not pilot tested, and thus was not validated prior to initiation of the study. The survey data provided information regarding complications but was likely subject to both response bias and non-response bias. Owners were also not asked if they had access to internet prior to enrolling in the study, which may have inadvertently biased recruitment to the study. Additionally, owner descriptions of skin complications were utilized to create the scoring system, with several responses labeled “unknown” due to lack of clear description. Owners were not provided with specific training in the categorization of complications, which likely contributed to the lack of clear descriptions and potentially increased the subjectiveness of the skin complication severity. The resulting scoring system was also subjective, despite two of the authors agreeing on scores for the provided descriptions. Due to the existence of the “unknown” label and the inability to determine the severity of these skin complications compared to those in the minor severity category, these categories were grouped together, which may not be an accurate representation of the true severity of the skin complications, as the “unknown” skin complication may have been more severe than those in the minor severity category. The reporting of the skin complications furthermore relied on each owner to detect these complications, which may have resulted in missed skin lesions and artificially low skin complications reported by less attentive owners.

Not all follow-up visits were utilized in data analysis, which may have resulted in missed trends in patient improvement or progressive lameness. This approach was selected due to the study's focus on long-term improvement and acclimation. In addition, the number and timing of follow-up visits were variable due to disturbance to clinical practice associated with COVID-19 pandemic restrictions and that several animals were euthanized prior to the completion of the study. Thus, the most recent follow-up visits occurred at different timepoints for various animals, ranging between 3 and 15 months. This resulted in the comparison of patient improvement at different stages in their disease process.

Further studies of each device with larger sample sizes and control groups are needed to objectively quantify whether these devices significantly improve lameness severity. Additionally, further studies are necessary to determine the specific implications of patient conformation with VOP. However, this study is the first that provides prospective data regarding complications and potential therapeutic benefit that can be utilized in daily clinical practice in the prescription of these devices.

## Data Availability Statement

The datasets presented in this study can be found in online repositories. The names of the repository/repositories and accession number(s) can be found in the article/supplementary material.

## Ethics Statement

The animal study was reviewed and approved by the Animal Care and Use Committee and Clinical Review Board of Colorado State University. Written informed consent was obtained from the owners for the participation of their animals in this study.

## Author contributions

SR and FD contributed to the conception and design of the study. SR created the initial online survey, which was revised and edited by FD. SR organized the data and wrote the first draft of the manuscript. All authors contributed to manuscript revision, read, and approved the submitted version.

## Conflict of interest

Author FD is a paid consultant of OrthoPets, LLC. The remaining authors declare that the research was conducted in the absence of any commercial or financial relationships that could be construed as a potential conflict of interest.

## Publisher's note

All claims expressed in this article are solely those of the authors and do not necessarily represent those of their affiliated organizations, or those of the publisher, the editors and the reviewers. Any product that may be evaluated in this article, or claim that may be made by its manufacturer, is not guaranteed or endorsed by the publisher.
