# Supplementary material for: Prospective evaluation of complications associated with orthosis and prosthesis use in canine patients
Source: Front Vet Sci. 2022 Jul 29;9:892662. doi: 10.3389/fvets.2022.892662 (PMC9372342; doi:10.3389/fvets.2022.892662)
Supplement: Supplementary file 1 [file Table_1.DOCX]

**Online Survey Questions** **and Answer Options** All questions were required to be answered by the owners apart from questions 11 and 12, which were only asked of the owner if they reported a skin complication in question 10. Questions 5-8 were answered with a slider bar with available options between 0-100.

Q1- During the last month, have there been any changes in your dog's health, environment or medication regime?

- Yes (please specify the changes)
- No, everything is unchanged

Q2- On average, during the last month, how often did your dog wear the brace on a daily basis?

- Not at all
- A few minutes per day
- A few hours per day
- Almost all day (but not at night)
- Almost all day and night

Q3- During the last month, did you adhere to the brace wearing schedule suggested by your veterinarian?

- Yes
- No, my dog wore the device more often than recommended
- No, my dog wore the device less often than recommended
- My veterinarian did not suggest a wearing schedule
- Other (please specify)

Q4- Has your dog received any form of rehabilitation (physical therapy) during the last month?

- Yes, our dog had at least one session with a rehabilitation (physical therapy) specialist and we have performed rehabilitation at home
- Yes, our dog had at least one session with a rehabilitation (physical therapy) specialist
- Yes, we have performed rehabilitation (physical therapy) ourselves at home
- No
- Other (please specify)

Q5- Overall, during the last month, how much do you think your dog benefited from the brace? (please use the slider below to select how beneficial the brace was for your dog)

Q6- Overall, during the last month, how active was your dog? (please use the slider below to select your dog's activity level)

Q7- Overall, during the last month, how happy was your dog? (please use the slider below to select your dog's happiness level)

Q8- Overall, during the last month, how satisfied are you with the brace as a treatment for your dog's disease? (please use the slider below to select your satisfaction level with the brace)

Q9- During the last month, have there been any complications (other than skin sores) associated with the brace?

- Yes (please specify the complication/s)
- No, everything is fine

Q10- During the last month, did your dog develop any skin sores, skin irritation, or other wounds from wearing the brace?

- Yes
- No

Q11- Please describe the skin sore, irritation, or wound.

Q12- Did you have your dog assessed by a veterinarian for the skin sore, irritation, or wound?

- Yes
- No
- I have not yet, but I plan to
- Other (please specify)
